# Hemorheology in Inflammatory Bowel Disease: A Case–Control Study

**DOI:** 10.3390/jcm14134436

**Published:** 2025-06-22

**Authors:** Zsolt Szakács, Beáta Csiszár, Mátyás Nagy, Margit Tőkés-Füzesi, Patrícia Sarlós, Kálmán Tóth, Péter Hegyi, Hussain Alizadeh, Judit Bajor

**Affiliations:** 1 First Department of Medicine, Medical School, University of Pécs, H-7624 Pécs, Hungary; sarlos.patricia@pte.hu (P.S.); toth.kalman@pte.hu (K.T.); alizadeh.hussain@pte.hu (H.A.); bajor.judit@pte.hu (J.B.); 2 Institute for Translational Medicine, Medical School, University of Pécs, H-7624 Pécs, Hungary; hegyi.peter@pte.hu; 3 Department of Anaesthesiology and Intesive Therapy, University of Pécs, H-7624 Pécs, Hungary; csiszar.beata@pte.hu; 4 Saint Raphael Hospital of Zala Castle County, H-8900 Zalaegerszeg, Hungary; nagymatyeee@gmail.com; 5 Department of Laboratory Medicine, Medical School, University of Pécs, H-7624 Pécs, Hungary; tokes-fuzesi.margit@pte.hu; 6 Szentagothai Research Centre, University of Pecs, H-7624 Pecs, Hungary; 7 Institute of Pancreatic Diseases, Semmelweis University, H-1083 Budapest, Hungary; 8 Centre for Translational Medicine, Semmelweis University, H-1085 Budapest, Hungary

**Keywords:** inflammatory bowel disease, Crohn’s disease, ulcerative colitis, viscosity, aggregation, thrombosis, anticoagulant, hemorheology

## Abstract

**Background**: Venous thromboembolism is more prevalent among patients with inflammatory bowel disease (IBD). This study aimed to identify prothrombotic hemorheological alterations in IBD. **Methods**: We conducted a case–control study with patients with ulcerative colitis, Crohn’s disease, and non-IBD control subjects. We measured hemorheological indicators including plasma viscosity (PV), whole blood viscosity (WBV), erythrocyte aggregation (EA), and erythrocyte deformability (ED). Uni- and multivariate tests were employed for analysis. **Results**: A total of 53 IBD patients and 77 control subjects were recruited. IBD patients showed significantly higher aggregation index (68.8% (35.3–83.5%) vs. 66.9% (35.2–83.5%), *p* = 0.003) and threshold shear rate (120 1/s (55–325 1/s) vs. 110 1/s (55–325 1/s), *p* < 0.001), with lower aggregation half-time (1.6 s (0.6–7.1 s) vs. 1.8 s (0.6–7.1 s), *p* = 0.004), indicating enhanced EA. However, after adjusting for covariates, including inflammatory markers, IBD no longer predicted EA. There were no significant differences in EA. PV, WBV, and ED between the groups. Fibrinogen, rather than the Crohn’s Disease Activity Index, was the strongest predictor of the outcomes. **Conclusions**: Our study demonstrates that IBD patients exhibit enhanced EA, predicted mainly by fibrinogen. These results confirm that inflammation plays the cardinal role in the increased tendency for venous thromboembolism in IBD.

## 1. Introduction

Crohn’s disease (CD) and ulcerative colitis (UC), the most prevalent forms of inflammatory bowel disease (IBD), share a common underlying feature: inflammation [1].

The association of inflammation and thrombus formation can be explained by overlapping molecular mechanisms, which have been corroborated by clinical observations of elevated thrombotic risk in various immune-mediated and autoimmune disorders, including IBD [2]. According to a meta-analysis involving adult IBD patients, the risk of venous thromboembolism (VTE), compared to the general population, is 2-fold (relative risk (RR) = 1.96, 95% confidence interval (CI): 1.67–2.30). In contrast, reports on arterial thrombotic events are less consistent; however, in non-hospitalized IBD patients, there was a notable 28% increase in arterial thrombotic events compared to the general population (RR = 1.28, 95% CI: 1.16–1.42). The risk of ischemic heart disease was significantly elevated (RR = 1.35, 95% CI: 1.19–1.52), as was the risk of mesenteric ischemia (RR = 3.46, 95% CI: 1.78–6.71). Conversely, the risk of stroke (RR = 0.79, 95% CI: 0.51–1.23) and peripheral arterial disease (RR = 0.78, 95% CI: 0.46–1.32) did not show a significant increase. Interestingly, cardiovascular mortality was not found to be higher in IBD compared to the general population (standardized mortality rate = 1.03, 95% CI: 0.93–1.14, showing similar figures for both UC and CD) [3]. Furthermore, a recent Mendelian randomization study did not find support for a causal relationship between IBD and coronary artery disease or ischemic stroke [4]. These findings suggest that the elevated thrombotic risk is mainly driven by VTE rather than arterial thrombotic events [3].

Virchow, who primarily focused on VTE, proposed that thrombus formation has three key factors: endothelial injury, stasis, and hypercoagulability [5]. Among these, hypercoagulability driven by inflammation is considered the leading cause of the elevated thrombotic risk in IBD. Intestinal dysbiosis and bacterial translocation are assumed to play a cardinal role as triggers in the process [6,7]. A recent review of Menichelli et al. proposes a model explaining a tendency for VTE: among others, alterations of the primary, secondary, and tertiary hemostasis, along with endothelial injury, may be involved [2]. Additionally, changes in blood rheology—such as in plasma viscosity (PV), whole blood viscosity (WBV), erythrocyte aggregation (EA), and erythrocyte deformability (ED)—could also contribute to hypercoagulability [8]. To enhance the readability of our paper, we provide a general overview of hemorheology-related terms and their interpretation in Table 1 [9].

**Table 1:** Hemorheology-related terms and measurements.

Parameter (Abbreviation)	Measurement (Abbreviation or Symbol, Unit)	Definition	Unfavorable Alteration ^a^
Erythrocyte deformability (ED)	Elongation index (EI, no unit)	Change in the shape of red blood cells at high (from EI30Pa to EI3Pa in this study) and low shear stresses (from EI3Pa to EI0.3Pa in this study) (shown in Figure 1 inlet)	↓
Erythrocyte aggregation (EA)	Aggregation index (AI, %)	Integral in the change in light intensity 10 s after disaggregation	↑
	Aggregation half-time (t1/2, s)	The time required for achieving half of the maximal aggregation after disaggregation	↓
	Threshold shear rate (γ, s^−1^)	Lowest shear that can maintain complete disaggregation	↑
Viscosity	Whole blood viscosity (WBV, mPa·s)	An intrinsic property of fluid related to the internal friction of adjacent fluid layers sliding past one another (i.e., the measure of a fluid’s resistance	↑
	Plasma viscosity (PV, mPa·s)		↑

^a^ Regarding thrombus formation. AI, t1/2, and γ were measured with Laser-assisted Optical Rotational Cell Analyzer (LORCA; R&R Mechatronics, Hoorn, the Netherlands), EI was measured with laser-diffraction ektacytometry with a LORCA, and WBV and PV were measured with Brookfield DV-III Ultra LV Programmable rotational viscometer (Brookfield Engineering Labs, Middleboro, MA) at mid-shear (90 s^−1^). The table is reproduced based on our previous open-access article in Clinical and Translational Gastroenterology [9].

**Figure 1:**
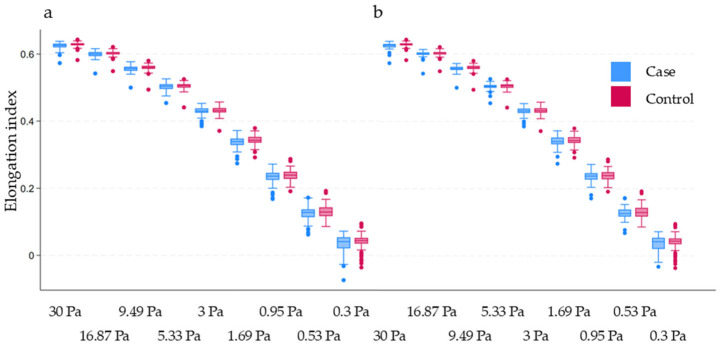
Erythrocyte deformability at different levels of shear stress. The horizontal axis represents shear stress. (**a**) Inflammatory bowel disease (case) vs. control; (**b**) Crohn’s disease (case) vs. control. The *p*-value is >0.0055 (after Bonferroni correction) in all cases, indicating no statistically significant difference between the groups.

In this case–control study, we aimed to achieve two primary objectives: (1) to test if prothrombotic hemorheological alterations are present in IBD and (2) to test if hemorheological parameters are influenced by the disease activity of IBD.

## 2. Results

Our study included 53 IBD patients and 77 control subjects. Among the IBD patients, 15 were diagnosed with UC and 38 with CD. The median age at diagnosis for IBD patients was 23 y (range: 9–60 y).

Table 2 and Table 3 provide a summary of the clinical and laboratory characteristics of the study population, respectively. In terms of clinical characteristics, IBD patients were statistically significantly younger (mean 30 vs. 44 y), more likely to be male (50.9% vs. 27.3%), and less likely to have lipid metabolism disorders in history, compared to the control subjects (Table 2). Regarding laboratory characteristics, IBD patients exhibited significantly elevated levels of alkaline phosphatase, C-reactive protein, fibrinogen, significantly higher white blood cell, neutrophil, and platelet counts, and a higher erythrocyte sedimentation rate, compared to the control group. Conversely, IBD patients had significantly lower levels of high-density and low-density lipoproteins, lactate-dehydrogenase, cholesterol, albumin, and hemoglobin; and a lower hematocrit value, compared to the control group (Table 3).

PV, WBV, and ED as measured by elongation indices (EI) at nine different levels of shear stress were not statistically significantly different between the groups (for EI at varying levels of shear stress, see Figure 1). In contrast, all the EA-related parameters were significantly different between groups, indicating a tendency for increased EA in IBD (Table 4). After adjustment for age and sex in Model A, IBD remained a significant predictor of aggregation index and threshold shear rate, although it was not a significant predictor of aggregation half-time. However, after adjustment for further relevant variables, including inflammatory markers in Model B, IBD was no longer a significant predictor of EA-related parameters. In this adjusted model, fibrinogen was the strongest predictor (Table 5).

Table 2 and Table 3 present a summary of the clinical and laboratory characteristics of CD patients, respectively. The associations observed in this subgroup mirrored those found in the comparison of IBD vs. the control groups, with an exception: CD was not a significant predictor of aggregation half-time, as shown in Table 4. In Model C, adjusting for age and sex did not alter the associations. However, after further adjustment with other relevant variables, including inflammatory markers in Model D, CD was no longer a significant predictor of EA-related parameters. Similar to the findings for IBD, fibrinogen was the strongest predictor (Table 5).

**Figure 2:**
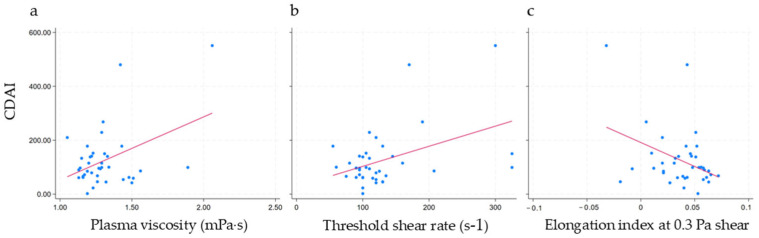
Linear regression of Crohn’s Disease Activity Index (CDAI) with study outcomes. (**a**) CDAI and plasma viscosity; (**b**) CDAI and threshold shear rate; (**c**) CDAI and elongation index at 0.3 Pa shear stress.

Upon examining the association with disease activity as measured by the Crohn’s Disease Activity Index, CDAI, we found a weak but statistically significant, positive correlation between CDAI and both PV and threshold shear rate. No other significant correlations were found (Figure 2 and Table 6). Adjusting for age and sex did not alter the associations. However, when fibrinogen was included in the model, the R^2^ value dramatically increased from 18% to 60% for PV and from 19% to 80% for threshold shear rate, indicating that fibrinogen accounted for a substantial portion of the variance. Still, CDAI remained a weak, statistically significant predictor of the outcome.

## 3. Discussion

In this case–control study, we aimed to assess the hemorheological profile of IBD patients. Our findings indicate that IBD patients exhibit enhanced EA, primarily driven by inflammation, while there were no significant alterations in PV, WBV, and ED. Additionally, we found a weak, positive correlation between CDAI and both PV and threshold shear rate.

We aimed to identify all human, original studies reporting on the role of viscosity, EA, and ED in IBD patients with a systematic search in MEDLINE (via PubMed). This search yielded six relevant studies.

To the best of our knowledge, the earliest publication on the role of hemorheology in IBD dates back to 1992. In this study, Lobo et al. measured PV with a Coulter viscometer in IBD patients (CD and UC, no control group) and reported some weak associations with disease activity [10]. In 1995, Moran et al. found a moderately strong, positive correlation between PV and endoscopic severity in a small group of IBD patients (CD and UC, no control group) [11].

In 1996, Novacek et al. published the first controlled study in CD, providing a detailed analysis of PV measured with a Coulter viscometer and, to our best knowledge, the first assessment of EA measured with a Myrenne aggregometer. Their findings revealed that fibrinogen, PV, and EA were significantly higher in patients with CD, compared to the control group. Although patients with active CD had higher numerical values of PV and EA compared to those with inactive CD, the differences did not reach statistical significance. Correlation analysis further confirmed the absence of a significant association between EA and disease activity [12].

In 2005, Zilberman et al. published a matched controlled study that assessed EA measured with a slide test. Their findings indicated an activity-dependent, enhanced EA in both CD and UC patients compared to the control group [13]. Another paper (Maharshak et al.) published in 2009 by the same research group reported on the measurement of EA with or without in vitro plasma exchange with a microscopic method in a controlled study (in CD and UC). Here, an enhanced disease activity-dependent EA was observed. Interestingly, when the patients’ plasma was removed, no significant differences were observed between the groups, suggesting that a plasma factor—potentially fibrinogen or other acute-phase reactant—was responsible for the enhanced EA [14,15].

In 2012, Akman et al. published a controlled study including CD and UC patients, marking the first to report on ED measured with Laser-assisted Optical Rotational Cell Analyzer (LORCA) in IBD. In this study, EI (the level of shear stress was not specified) was the lowest in patients with active IBD, followed by those with inactive IBD, then by the control subjects, suggesting an activity-dependent impairment of ED. ED did not correlate with measures of oxidative stress [16].

In 2016, an Italian-language paper by Yakar et al. reported PV to be a useful marker in predicting therapeutic response to glucocorticoids or cyclosporin in UC patients [17].

To summarize the existing literature, a limited number of previous studies have revealed inconsistent findings on PV and rather prothrombotic alterations of EA and ED in IBD. In the following paragraphs, we summarize our findings to contrast with previous evidence.

- PV: We found no significant difference between the groups, and the correlation with CDAI was also weak (and it did not explain much of the variance).
- WBV: We found no significant difference between the groups. To our best knowledge, WBV has not been examined in this context before.
- EA: We observed significant prothrombotic alterations of EA-related parameters in IBD and CD. Previous studies measured EA with the Myrenne aggregometer, with a slide test, and with microscopy, while we measured EA with the LORCA, allowing for a more detailed assessment. However, it is important to note that the magnitude of differences between the groups was not substantial, and their clinical implications require further investigations to draw more reliable conclusions regarding their role.
- ED: We found no significant difference between the groups after the Bonferroni adjustment for multiplicity. This finding contradicts an earlier one about activity-dependent prothrombotic changes. However, our study employed a LORCA-based method on varying levels of shear stress, whereas in the previous study, only one level of shear stress was used.
- The role of inflammatory markers: Previous reports have concluded that the inflammatory response and (directly or indirectly) the acute-phase reactants were responsible for the prothrombotic hemorheological changes. Our results support this hypothesis as fibrinogen proved to be the best predictor of the altered EA. Hypothetically, this suggests that the more active the disease, the more pronounced prothrombotic alterations are observed.

The main finding of our study is the identified association between EA and IBD. EA is an important pathophysiological determinant of thrombus formation, particularly at a low level of shear stress in the venous system. Several mechanisms have been proposed to explain this: increased EA may facilitate platelet migration [18], impair blood flow of the microcirculation [19], and contribute to aggregate formation [20,21]. From a clinical perspective, previous studies showed that patients with deep vein thrombosis exhibit an increased EA (for a comprehensive review, see the paper of Vajá et al.) [22]. However, it is essential to note that in our study, while the numerical differences between the groups were statistically significant for most EA-related parameters, they were not particularly pronounced. Additionally, there is limited data available regarding the precise interpretation of EA-related parameters in relation to the risk of VTE. This highlights the need for further research to better understand the clinical implications of EA in IBD patients.

This study has several strengths that enhance the robustness of our findings: (1) We provide a comprehensive evaluation of the hemorheological profile of IBD patients, integrating both clinical and laboratory evidence. (2) We aimed to balance confounding factors between the groups by performing multivariate analysis, which was made possible by our adequate sample size. (3) The careful selection of the control subjects and the strict eligibility criteria of our study aimed to minimize selection bias. (4) We performed standardized hemorheological measurements by strict laboratory rules to increase accuracy and reproducibility.

This study has some limitations. (1) Due to clinical resource constraints, the number of patients with UC was limited and did not allow us to perform subgroup analysis. The majority (38 of 53 patients) of the IBD population had CD. Consequently, our results may be heavily influenced by this subgroup. (2) We strove to analyze the role of inflammation and disease activity in triggering prothrombotic alterations, but we did not account for the effect of IBD medications. Modern targeted anti-IBD medications—such as small molecules and biological therapeutics—may mitigate the risk of VTE by reducing inflammation. Future studies should investigate the potential impact of these anti-IBD medications on hemorheological indicators, as well as the effects of other drugs, including platelet inhibitors, antihypertensive agents, and antioxidants. (3) The case–control study design is not adequate to confirm a cause–effect relationship due to bias inherent in observational studies. A cohort study with follow-up and re-testing could better approximate causality. (4) Although we employed multivariate statistical analysis to overcome the effect of confounding factors, hemorheology is influenced by many covariates that are still unknown, were not captured by this study, or that we could not use in the analysis due to technical reasons. (5) The control group included medical personnel and their relatives, which may introduce selection bias, as these individuals might be healthier than the general population.

## 4. Materials and Methods

The study was registered in the ISRCTN Registry under registration number ISRCTN49677481, and detailed methodological information can be found in the pre-study protocol [23]. The study has three arms: IBD, celiac disease, and non-celiac, non-IBD control groups. This paper reports only on the IBD and the control arms, whereas the findings of celiac vs. control comparison are reported elsewhere [9].

### 4.1. Design, Setting, and Eligibility

This is a single-center case–control study.

Inclusion criteria (applies to all subjects):

- Blood collection must be indicated with medical conditions.
- Signed informed consent.

Inclusion criteria (applies to specific cohorts of patients):

- IBD patients: newly diagnosed or follow-up patients (with active or remitting disease) aged ≥18 years (not following a gluten-free diet); the establishment of a diagnosis should meet the current guidelines (European Crohn’s and Colitis Organisation (ECCO) (available online at https://www.ecco-ibd.eu/ accessed on 1 January 2017).
- Non-celiac, non-IBD control subjects: individuals aged ≥18 years (not following a gluten-free diet) in whom celiac disease and IBD can be excluded according to the recent guidelines.

Exclusion criteria (applies to all subjects):

- Chronic conditions:
  - Estimated glomerular filtration rate calculated with the Chronic Kidney Disease Epidemiology Collaboration (CKD-EPI) formula is <60 mL/min/1.73 m^2^ (CKD3 or more severe kidney failure).
  - Liver cirrhosis in Child–Pugh B–C.
  - Heart failure (New York Heart Association (NYHA) III–IV).
  - Active malignant diseases.
- Any acute diseases or invasive procedures within 2 weeks of recruitment (eg, systemic infection, surgery, or major trauma).
- Thrombotic events within 1 year of recruitment.
- Ongoing oral anticoagulant therapy (vitamin K antagonists) and/or antiplatelet drugs.
- Confirmed systemic lupus erythematosus.
- Pregnancy.
- Patients are unable to understand the essentials of informed consent.

Subjects were recruited consecutively over a year between June 2018 and May 2019. Control subjects were recruited from our gastroenterology outpatient clinic, or were volunteer medical personnel or their relatives.

Further details are provided in the pre-study protocol [23].

### 4.2. Flow and Timing

After obtaining informed consent, a medical doctor recorded each subject’s medical history, which included a detailed thrombophilia-related assessment and signs and symptoms. This was followed by blood collection in the central laboratory unit of our center. It is important to note that there was no follow-up conducted in this study.

### 4.3. Laboratory Measurements and Disease Activity

All measurements were performed in the laboratories of the University of Pécs. The measurements included routine laboratory studies (including but not limited to blood counts and inflammatory markers), antiphospholipid syndrome- and coeliac-specific immunological indicators, and screening hemostatic parameters (prothrombin time, thrombin time, activated partial thrombin time, fibrinogen). Laboratory personnel were blinded to clinical data, while the enrolling physician was blinded to the laboratory data. Further details are provided in the pre-study protocol [23].

The measurements related to hemorheology included EA determined with LORCA (R&R Mechatronics, Hoorn, The Netherlands), ED with laser-diffraction ektacytometry with a LORCA, and WBV and PV with Brookfield DV-III Ultra LV Programmable rotational viscometer (Brookfield Engineering Labs; Middleboro, MA, USA). When handling the samples, we adhered to the recommendations of the International Expert Panel for Standardisation of Haemorheological Methods [24].

Activity of UC was estimated with the modified Mayo Score and that of CD with CDAI [25,26].

### 4.4. Study Outcomes

The outcomes included PV, WBV, EA, and ED-related parameters.

### 4.5. Sample Size, Data, and Analysis

We initially planned to recruit 100 study participants, with 50 assigned to each arm, then we planned to recalculate the required number of patients to achieve sufficient power. However, in PV, WBV, and EA-related parameters, the difference between the groups was so small that further recruitment seemed unfeasible and unlikely to change the conclusions of the study. We decided to continue recruiting control subjects to enhance our ability to adjust for confounding factors in EA-related measurements.

The data collection process is described in detail in the pre-study protocol [23].

The statistical analysis was performed with StataNow/BE 18.5 (StataCrop, College Station, Brazos County, Texas, the USA). In the descriptive analysis, continuous variables were summarized using mean or median with dispersion measures (depending on distribution), while categorical variables were expressed as proportions. In the comparative univariate analysis, we used the two-sample t-test or the Mann-Whitney test for continuous variables (adjusted for multiplicity by Bonferroni if needed), and the chi^2^-test or Fisher’s exact test for categorical variables. When building multivariate models, we selected potential predictors by using linear correlation and clinical judgement of relevance. In multivariate analysis, we used multiple linear regression (with the assessment of multicollinearity).

## 5. Conclusions

Our study shows that IBD patients, including CD patients, exhibit enhanced EA driven primarily by inflammation, particularly through elevated fibrinogen levels. In contrast, PV, WBV, and ED do not appear to be affected significantly by the disease. While demonstrating modest predictive strength, the CDAI showed statistically significant positive associations with PV and, to a lesser extent, with EA. These findings confirm that inflammation plays the cardinal role in the increased tendency for VTE in IBD. Further studies are needed to test whether pharmacological interventions can restore the normal thrombotic milieu in IBD. Prospective longitudinal studies could further clarify the role of hemorheology in thrombotic risk stratification in IBD.

## Figures and Tables

**Table 2:** Clinical characteristics of the study population.

Variables	Inflammatory Bowel Disease (*n* = 53)	Crohn’s Disease (*n* = 38)	Control Group (*n* = 77)	Inflammatory Bowel Disease vs. Control (*p*-Value)	Crohn’s Disease vs. Control (*p*-Value)
Age at study entry (years)	30 (18–79)	30 (18–65)	44 (19–75)	**0.027**	**0.016**
Sex, n (female%)	26 (49.1)	19 (50.0)	56 (72.7)	**0.006**	**0.016**
Body mass index (kg/m^2^)	24.2 (5.3)	23.8 (5.4)	24.4 (3.9)	0.823	0.494
Venous thrombosis in history	1 (1.9)	1 (2.6)	1 (1.3)	N/I	N/I
Arterial thrombosis in history	0 (0.0)	0 (0.0)	1 (1.3)	N/I	N/I
Family history positive for any thrombosis	15 (28.3)	13 (34.2)	20 (26.0)	0.769	0.358
Ongoing hormone replacement therapy	0 (0.0)	0 (0.0)	1 (1.3)	N/I	N/I
Ongoing oral contraceptive use, n (% of females)	4 (15.4)	3 (15.8)	12 (21.4)	0.765	0.747
Current smoker	12 (22.6)	12 (31.6)	9 (11.7)	0.078	**0.009**
Regular alcohol consumption	2 (3.8)	1 (2.6)	3 (3.9)	N/I	N/I
Malignancy in history	1 (1.9)	1 (2.6)	1 (1.3)	N/I	N/I
Major trauma, plaster cast in the past 3 months	0 (0.0)	0 (0.0)	1 (1.3)	N/I	N/I
Immobilization (>3 days bedrest)	0 (0.0)	0 (0.0)	1 (1.3)	N/I	N/I
Traveling a long distance (>6 h in sitting position)	4 (7.6)	2 (5.2)	5 (6.5)	N/I	N/I
Any varicose veins	11 (20.8)	8 (21.1)	16 (20.8)	1.000	1.000
Peripheral arterial disease in history	1 (1.9)	1 (2.6)	2 (2.6)	N/I	N/I
Surgery in the past 2 weeks	4 (7.6)	3 (7.9)	10 (13.0)	0.398	0.540
Stroke or acute myocardial infarction in history	0 (0.0)	0 (0.0)	0 (0.0)	N/I	N/I
Hypertension in history	6 (11.3)	2 (5.2)	18 (23.4)	0.082	**0.018**
Diabetes mellitus in history	2 (3.8)	0 (0.0)	7 (9.1)	0.309	0.094
Lipid metabolism disorder in history	6 (11.3)	2 (5.2)	21 (27.3)	**0.028**	**0.006**

For continuous variables, central tendency and measure of dispersion are reported, as follows: median (range) or mean (SD). For categorical variables, n and % of total are reported if not indicated otherwise in the table. NI: not interpretable due to the low event number. IBD: inflammatory bowel disease; CD: Crohn’s disease. Bold highlight indicate a *p*-value < 0.05.

**Table 3:** Laboratory characteristics of the study population.

Variables	Inflammatory Bowel Disease (*n* = 53)	Crohn’s Disease (*n* = 38)	Control Group (*n* = 77)	Inflammatory Bowel Disease vs. Control (*p*-Value)	Crohn’s Disease vs. Control (*p*-Value)
Blood urea nitrogen (mmol/L)	4.2 (1.3)	4.0 (1.1)	4.6 (1.3)	0.126	**0.036**
Creatinine (μmol/L)	79.0 (22.3)	76.6 (16.2)	73.9 (13.7)	0.109	0.349
Aspartate aminotransferase (U/L)	23 (10)	23 (10)	24 (21)	0.836	0.815
Alanine aminotransferase (U/L)	25 (22)	24 (23)	24 (27)	0.885	0.936
Alkaline phosphatase (U/L)	86 (40)	85 (38)	68 (17)	**<0.001**	**<0.001**
γ-glutamyl carboxylase (U/L)	36 (74)	27 (28)	25 (29)	0.241	0.667
Bilirubin (umol/L)	9.1 (5.9)	9.4 (6.5)	10.4 (8.1)	0.340	0.517
High-density lipoprotein cholesterol (mmol/L)	1.5 (0.5)	1.5 (0.5)	1.7 (0.5)	**0.019**	**0.038**
Non-high-density lipoprotein cholesterol (mmol/L)	3.1 (1.1)	2.8(0.8)	3.5 (1.1)	0.069	**0.001**
Low-density lipoprotein cholesterol (mmol/L)	3.0 (1.1)	2.7 (0.9)	3.5 (1.1)	**0.019**	**<0.001**
Triglyceride (mmol/L)	1.5 (1.3)	1.5 (0.8)	1.7 (1.2)	0.455	0.349
Total protein (g/L)	75.5 (5.3)	74.7 (5.2)	75.1 (4.3)	0.692	0.667
Albumin (g/L)	47.8 (3.4)	47.5 (3.8)	49.3 (3.4)	**0.017**	**0.019**
Ultrasensitive C-reactive protein (mg/L)	8.1 (15.5)	9.3 (17.9)	2.2 (2.4)	**0.001**	**<0.001**
Prothrombin time (s)	11.6 (1.2)	11.4 (1.0)	11.2 (0.7)	**0.032**	0.118
Thrombin time (s)	14.0 (1.0)	13.9 (1.1)	14.2 (1.0)	0.152	0.088
Activated partial thromboplastin time (s)	30.9 (4.6)	31.0 (5.0)	29.5 (6.4)	0.174	0.193
Fibrinogen (g/L)	3.6 (1.1)	3.6 (1.2)	3.1 (0.6)	**0.002**	**0.004**
White blood cell (G/L)	8.2 (5.4)	8.6 (6.1)	6.8 (1.9)	**0.038**	**0.019**
Neutrophil granulocyte (G/L)	5.7 (5.2)	6.2 (6.0)	4.0 (1.5)	**0.009**	**0.003**
Hemoglobin (g/L)	136 (17)	138 (18)	143 (13)	**0.008**	0.081
Hematocrit (%)	39.8 (4.3)	40.2 (4.7)	41.5 (3.5)	**0.014**	0.097
Mean corpuscular volume (fL)	86 (8)	88 (8)	85 (4)	0.364	0.064
Platelet (G/L)	321 (115)	308 (123)	276 (66)	**0.006**	0.069
Erythrocyte sedimentation rate (mm/h)	13 (11)	13 (12)	6 (5)	**<0.001**	**<0.001**

For continuous variables, mean (SD) is reported. In this table, hematocrit was estimated with automated cell counter. IBD: inflammatory bowel disease; CD: Crohn’s disease. Bold highlight indicate a *p*-value < 0.05.

**Table 4:** The hemorheological profile.

Variables	IBD Patients (*n* = 53)	Crohn’s Disease (*n* = 38)	Control Subjects (*n* = 77)	IBD vs. Control (*p*-Value)	Crohn’s Disease vs. Control (*p*-Value)
Plasma viscosity (mPa·s)	1.31 (0.18)	1.31 (0.20)	1.27 (0.13)	0.141	0.180
Whole blood viscosity (mPa·s)	4.11 (0.53)	4.14 (0.59)	4.11 (0.45)	0.958	0.779
Hematocrit (%)	42.6 (4.5)	43.0 (5.0)	44.2 (3.4)	**0.023**	0.112
Aggregation index (%)	68.8 (35.3–83.5)	66.9 (35.2–83.5)	66.1 (47.1–75.8)	**0.003**	**0.048**
Aggregation half-time (s)	1.6 (0.6–7.1)	1.8 (0.6–7.1)	1.8 (1.1–4.5)	**0.004**	0.059
Threshold shear rate (s^−1^)	120 (55–325)	110 (55–325)	100 (50–192.5)	**<0.001**	**0.007**

For continuous variables, central tendency and measure of dispersion are reported, as follows: median (range) or mean (SD). Bold highlight indicate a *p*-value < 0.05.

**Table 5:** Multivariate regression analysis of erythrocyte aggregation-related parameters.

	Aggregation Index		Aggregation Half-Time		Threshold Shear Rate	
	Coeff	*p*-Value	Coeff	*p*-Value	Coeff	*p*-Value
**Model A**						
IBD vs. control	4.0	**0.004**	−0.3	0.068	37.1	**<0.001**
Age	0.1	0.119	−0.01	0.210	0.4	0.098
Sex	0.7	0.635	−0.03	0.846	19.7	**0.012**
**Model B**						
IBD vs. control	1.8	0.101	−0.2	0.264	0.2	0.963
Age	0.1	0.082	−0.01	0.179	0.1	0.414
Sex	0.7	0.643	−0.1	0.473	−8.5	0.229
Creatinine	−0.02	0.515	0.00	0.487	−0.01	0.912
Bilirubin	−0.1	**0.029**	0.02	**0.003**	−0.21	0.441
Non-high-density lipoprotein cholesterol	0.4	0.374	−0.05	0.324	1.1	0.571
Total protein	0.3	**0.002**	−0.04	**0.004**	1.0	**0.044**
C-reactive protein	−0.2	**0.016**	0.02	**0.009**	1.6	**<0.001**
Fibrinogen	4.0	**<0.001**	−0.5	**<0.001**	18.7	**<0.001**
White blood cell count	−0.04	0.735	0.01	0.666	−1.2	**0.047**
Hematocrit	0.6	**<0.001**	−0.09	**<0.001**	−2.5	**0.002**
Platelet count	0.01	0.072	−0.00	0.169	−0.02	0.556
Erythrocyte sedimentation rate	0.3	**0.004**	−0.03	**0.021**	1.2	**0.016**
**Model C**						
CD vs. control	−3.5	**0.022**	0.2	0.183	−39.4	**<0.001**
Age	0.07	0.100	−0.01	0.235	0.6	**0.026**
Sex	0.1	0.935	0.04	0.829	18.2	**0.031**
**Model D**						
CD vs. control	−1.1	0.345	0.1	0.524	−2.8	0.632
Age	0.04	0.286	−0.00	0.543	0.3	0.077
Sex	0.2	0.897	−0.1	0.565	−12.5	0.113
Creatinine	0.03	0.525	0.00	0.619	−0.1	0.674
Bilirubin	−0.1	**0.044**	0.02	**0.007**	−0.35	0.222
Non-high-density lipoprotein cholesterol	0.05	0.920	−0.02	0.693	1.9	0.373
Total protein	0.3	**0.034**	−0.03	0.066	0.9	0.125
C-reactive protein	−0.2	**0.012**	0.02	**0.017**	1.8	**<0.001**
Fibrinogen	4.1	**<0.001**	−0.47	**<0.001**	16.7	**0.001**
White blood cell count	0.09	0.496	0.01	0.452	−1.2	0.061
Hematocrit	0.6	**<0.001**	−0.1	**<0.001**	−2.7	**0.002**
Platelet count	0.01	0.150	−0.00	0.255	−0.02	0.630
Erythrocyte sedimentation rate	0.4	**0.002**	−0.03	0.017	1.0	0.074

In these analyses, hematocrit was calculated with the capillary method. IBD: inflammatory bowel disease; CD: Crohn’s disease. Bold highlight indicate a *p*-value < 0.05.

**Table 6:** Univariate linear regression analysis between the hemorheological indicators and Crohn’s Disease Activity Index.

Variables	Regression Coeff	R^2^	*p*-Value
Plasma viscosity	0.001	0.18	**0.008**
Whole blood viscosity	−0.001	0.06	0.137
Aggregation index	0.004	<0.01	0.750
Aggregation half-time	−0.001 (<)	<0.01	0.967
Threshold shear rate	0.251	0.19	**0.007**
Elongation index at 30 Pa	−0.001 (<)	<0.01	0.688
Elongation index at 16.87 Pa	−0.001 (<)	<0.01	0.739
Elongation index at 9.49 Pa	−0.001 (<)	<0.01	0.680
Elongation index at 5.33 Pa	−0.001 (<)	0.02	0.451
Elongation index at 3 Pa	−0.001 (<)	0.04	0.244
Elongation index at 1.69 Pa	−0.001 (<)	0.07	0.120
Elongation index at 0.95 Pa	−0.001 (<)	0.09	0.063
Elongation index at 0.53 Pa	−0.001 (<)	0.12	0.032
Elongation index at 0.3 Pa	−0.001 (<)	0.15	0.017

For erythrocyte deformability, the *p*-value is >0.0055 (after Bonferroni correction) in all cases, indicating no statistically significant difference between the groups. Bold highlight indicate a *p*-value < 0.05.

## Data Availability

Relevant data are available in the manuscript.

## Abbreviations

The following abbreviations are used in this manuscript:

CDAI	Crohn’s Disease Activity Index
CI	confidence interval
EA	erythrocyte aggregation
ECCO	European Crohn’s and Colitis Organisation
ED	erythrocyte deformability
EI	elongation index
IBD	inflammatory bowel disease
LORCA	Laser-assisted Optical Rotational Cell Analyzer
PV	plasma viscosity
RR	relative risk
UC	ulcerative colitis
VTE	venous thromboembolism
WBV	whole blood viscosity
